# Insights on the role of thyroid hormone transport in neurosensory organs and implication for the Allan–Herndon–Dudley syndrome

**DOI:** 10.1530/ETJ-23-0241

**Published:** 2024-03-19

**Authors:** Ángel García-Aldea, Marina Guillén-Yunta, Víctor Valcárcel-Hernández, Ana Montero-Pedrazuela, Ana Guadaño-Ferraz, Soledad Bárez-López

**Affiliations:** 1 Instituto de Investigaciones Biomédicas Sols-Morreale, Consejo Superior de Investigaciones Científicas (CSIC)-Universidad Autónoma de Madrid (UAM), Madrid, Spain

**Keywords:** thyroid hormone, MCT8 deficiency, neurosensory systems, audition, vision

## Abstract

Thyroid hormones play an important role during the development and functioning of the different sensory systems. In order to exert their actions, thyroid hormones need to access their target cells through transmembrane transporter proteins, among which the monocarboxylate transporter 8 (MCT8) stands out for its pathophysiological relevance. Mutations in the gene encoding for MCT8 lead to the Allan–Herndon–Dudley syndrome (AHDS), a rare disease characterised by severe neuromotor and cognitive impairments. The impact of MCT8 deficiency in the neurosensory capacity of AHDS patients is less clear, with only a few patients displaying visual and auditory impairments. In this review we aim to gather data from different animal models regarding thyroid hormone transport and action in the different neurosensory systems that could aid to identify potential neurosensorial alterations in MCT8-deficient patients.

## Introduction

Thyroid hormones (THs), T3 (3,5,3′-triiodo-l-thyronine), and T4 (3,5,3′,5′-tetraiodo-l-thyronine) are essential for the correct development and functioning of most organs. The thyroid gland mostly secretes T4, which is metabolised into the genomically active form T3 by the action of the enzymes deiodinase type 1 (DIO1) and type 2 (DIO2). On the other hand, the DIO3 deiodinates T4 and T3 into their inactive forms rT3 (reverse 3,3′,5′-triiodo-l-thyronine) and T2 (3,5-diiodo-l-thyronine), respectively, to terminate TH action. Once they reach their target tissue, T3 and T4 enter into their target cells through specific plasma membrane transporters. There are several transporter proteins with the capacity of transporting TH, including l-type amino acid transporters such as LAT1 and LAT2, the organic anion transporter1 C1 (OATP1C1), and the monocarboxylate transporters 8 (MCT8) and 10 (MCT10). Among these, MCT8 stands out for its physiological relevance (1). In the canonical pathway for TH action, T3 modulates gene transcription by binding to TH receptors (TRs), that include TRα1 (encoded by *THRA*), TRβ1, and TRβ2 (both encoded by *TRHB*). These nuclear receptors act as ligand modulated transcription factors that regulate the expression of different target genes. This is the pathway by which TH mediates most of its actions (2).

The Allan–Herndon–Dudley syndrome (AHDS), or MCT8 deficiency, is a rare X-linked disease caused by inactivating mutations in the *SLC16A2* gene, which encodes for MCT8 (3, 4, 5). Patients present severe neurological disorders that include delayed neurological development, severe intellectual disability, and central hypotonia with spastic paraplegia, among others. These alterations arise as a consequence of brain hypothyroidism (6, 7, 8), most probably caused by the lack of a functional MCT8 in the brain barriers impairing TH transport into the brain (9, 10, 11). In addition, AHDS is characterised by high serum concentrations of T3, low T4, and rT3, and normal or slightly high levels of thyroid-stimulating hormone (TSH), indicating that there are also alterations in TH synthesis and metabolism (12). The restricted TH entry to the brain and the subsequent dysregulation of the hypothalamus–pituitary–thyroid gland axis, in addition to impairments in TH metabolism might be responsible for an increase in circulating T3 levels (1), producing a state of peripheral hyperthyroidism. Thereby, AHDS encompasses with both a neurological and an endocrine component.

Recent discoveries suggest that the symptomatic spectrum of AHDS patients might be wider than initially expected. Some of the patients diagnosed in the last years present less severe clinical symptoms including mild intellectual disability and mild or non-existent extrapyramidal symptoms, and they even develop the ability to articulate simple sentences or stand by themselves (13, 14). This complex and heterogeneous symptomatology makes the diagnosis of AHDS even more challenging and suggests that different patients might benefit from different therapeutic approaches. In view of this, it might be of relevance to gather information on other previously overlooked symptoms that may serve as additional biomarkers to address the phenotypical complexity of the AHDS. In view of the limited data available regarding neurosensorial alterations in AHDS patients or on the role of TH in sensory pathways in humans, in the present review we have gathered data obtained from different animal models regarding TH transport on neurosensory pathways. To better contextualise the relevance of MCT8 in these sensory pathways, other regulators of TH availability and action are also discussed.

## Audition

The development of auditory performance is critically dependent on TH signalling. Hearing loss in humans has been associated with iodine deficiency and congenital hypothyroidism (15, 16). Audition takes place in the cochlea, which is the hearing organ located at the inner ear. This fluid-filled, spiral-shaped tube consists of three compartments: scala tympani, scala media and scala vestibuli. Separating the scala media and the scala tympani is the basilar membrane (BM) upon which lays the auditory sensory epithelium: the organ of Corti. The organ of Corti comprises a single row of inner hair cells (IHCs), which are the primary sensory receptors and an additional three rows of outer hair cells (OHCs). Overlying the organ of Corti is the tectorial membrane (TM), an extracellular matrix structure to which the stereocilia of the hair cells attach. Sound waves generate movements in the cochlear fluids, producing vibrations on the BM that are translated into radial displacements of the hair cells, culminating in the activation of mechanoelectrical transducer ion channels (17).

In rodents, the crucial period for TH action during the ontogeny of the main auditory cell types spans from late embryonic to neonatal stages (18, 19). Auditory defects and impaired cochlear differentiation have been observed as a result of developmental hypothyroidism (18, 20), as well as a result of deletions in TRs (21, 22, 23), deiodinases (24, 25) and TH transporters (26).

The genes encoding the TRα1 and TRβ TH receptors,* Thra* and *Thrb*, are expressed at the cochlea. In the rat, *Thra* has been found distributed throughout the entire cochlea, while *Thrb* has been predominantly observed at the organ of Corti (27, 28). Studies in *Thrb-*deficient mice indicated that the hearing impairments in these animals reside within the cochlea (29). Even though no apparent structural defects were initially observed in the cochlea of *Thrb*-deficient mice (29), it was later proposed that malformations of the TM, especially during early postnatal development, lead to reduced mechanical performance of the cochlea and deafness (21, 30). While mice with TRα deletions exhibit normal hearing (31), TRα1 in *Thrb-*deficient mice may substitute for the absence of TRβ (32), suggesting a role for TRα in audition. Interestingly, recent studies have found that mice with frameshift mutations in *Thra* are prone to age-related hearing loss and display some defects in OHC (33). According to this, deletions in both TRβ and TRα1 lead to delayed sensory epithelium differentiation, malformation of TM, impairment of electromechanical transduction in OHCs, and a low endocochlear potential (21). Of interest, the *Thra* and *Thrb* genes have also been found at the developing ossicles of the mouse middle ear, which allow sound transmission from the outer to the inner ear (34), suggesting a role for TH in the development of this structure. Supporting this idea, mice expressing a dominant-negative TRα1 protein (*Thra*^+/PV^ mice) present deafness and a range of middle ear abnormalities including defective ossicles (34).

Activity of deiodinases is also involved in auditory function. DIO2-deficient mice present hearing impairments (24, 35) that, interestingly, resemble those present in TRβ/TRα1-deficient mice. These alterations include delayed differentiation of the cochlear inner sulcus and sensory epithelium, and malformations in the TM (24). Due to the similarity in the hearing phenotype between TRβ/TRα1-deficient and DIO2-deficient mice, it has been proposed that DIO2 controls T3 availability that activates TRs in the cochlea (24). DIO3-deficient mice also present auditory deficits. Interestingly, expression of *Dio3* in the immature cochlea has been found to overlap with *Thrb* expression and precedes *Dio2* expression. In contrast to DIO2-deficient mice, DIO3-deficient animals display accelerated cochlear differentiation, all of which suggest that DIO3 prevents premature stimulation of TRβ (25).

To identify the role of TH transporters in auditory function, the expression of main TH transporters was explored during cochlear development between embryonic day (E) 18 and postnatal day (P) 15 (36). LAT1 was the main TH transporter identified in cochlear blood vessels at the spiral ganglion, spiral limbus, spiral ligament, and stria vascularis, suggesting a prominent role for LAT1 in the uptake of TH from the blood. LAT1 was also observed in the cell membrane of hair cells, both in IHC and OHCs. *Mct8* expression was prominently detected in the spiral ganglion, columnar cells in the greater epithelial ridge, and stria vascularis, while showing comparatively lower levels of expression in the spiral ligament, spiral limbus, and lesser epithelial ridge. *Mct8* expression peaked from P3 to P5, although was still present at the spiral ganglion, faintly at blood vessels of the stria vascularis, and the spiral ligament until at least P15. In the spiral ganglion and greater epithelial ridge, the *Mct8* expression was found overlapping with *Thrb* expression in the sensory epithelium. Expression of *Oatp1c1*, which in the rodent brain is usually found in microvessels, was observed in fibrocytes – a supporting cell type involved in regulating endolymph electrolyte homeostasis and immune response (37) – of the spiral limbus and spiral ligament close to blood vessels. Interestingly, this expression pattern was overlapping with *Dio2* expression, as previously described (38). *Mct10* was identified in a highly restricted pattern mainly at the outer sulcus epithelium. Further studies evidenced that, while MCT8- or MCT10-deficient mice do not present hearing impairments, double MCT8/MCT10-deficient mice displayed hearing loss. Sharlin *et al.* (26) found degeneration of hair cells, retarded cochlear remodelling and malformations in the TM in MCT8/MCT10-deficient mice, similar to findings in TRβ/TRα1-deficient mice. The findings suggest that, in addition to the systemic availability of TH, cochlear development and hearing also depend on the uptake or efflux of TH by transmembrane transporters at target cells. The TH transporter LAT2 seems to have a relevant role in the adult inner ear, as its expression – mainly present in fibrocytes of the spiral ligament and limbus – increases from P60 to P360 in mice (39). Consequently, LAT2-deficient mice present important alterations in several cochlear structures and age-related hearing loss (39). However, whether these effects are due to impaired TH action or defective transport of neutral amino acids is yet to be determined.

**Figure 1:**
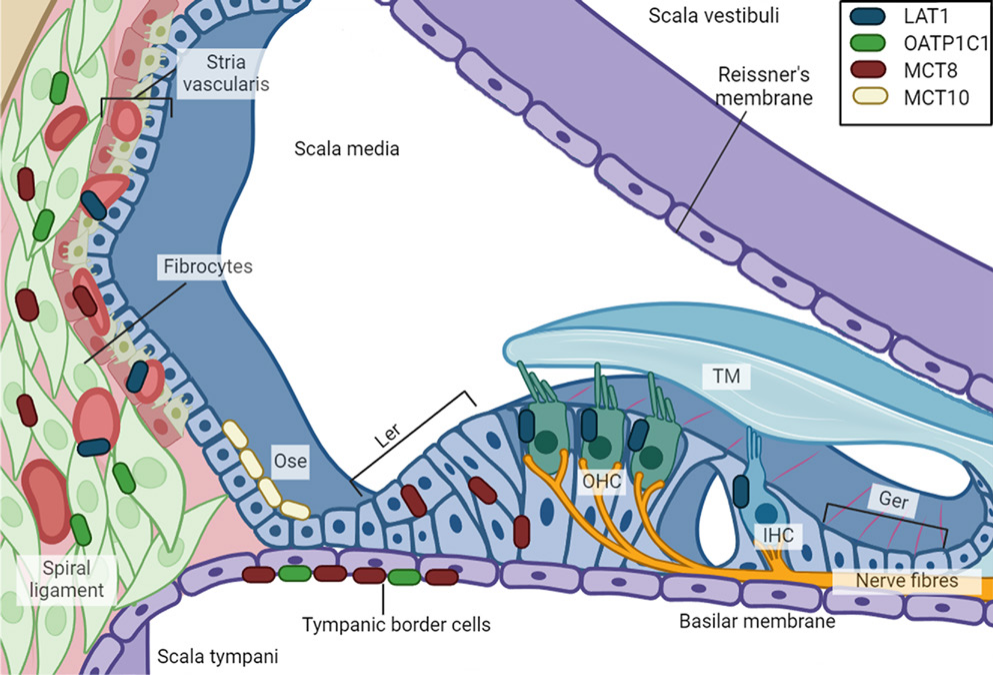
Proposed location of thyroid hormone (TH) transporters in the cochlea based on current findings. Findings from Sharlin *et al.* (36) identified *Lat1* expression in cochlear blood vessels at the spiral ganglion (not shown), spiral limbus (not shown), spiral ligament, and stria vascularis between P1 and P15; as well as in inner hair cells (IHCs) and outer hair cells (OHCs) at P3. *Mct8* expression was found in the greater epithelial ridge (Ger) and lesser epithelial ridge (Ler) at P1-P5; at the fibrocytes of the spiral ligament at P1-P15; at the tympanic border cells (tbc) a P7-P15; at the spiral ganglion neurons (not shown) at P1-P15; and faintly identified at blood vessels of the stria vascularis and the spiral ligament (not shown) at P7-P15. *Mct10* expression was found mainly restricted to the outer sulcus epithelium (ose) at P3-P7. *Oatp1c1* expression was observed at fibrocytes of the spiral ligament and the spiral limbus (not shown) and at the tbc at P7-P15.

Based on these findings, a model for the location of TH transporters in the inner ear is proposed in Fig. 1. Bearing in mind the limitations associated to determining the precise localisation of TH transporters as discussed below, we consider that the next challenge would be to assess if TH uptake from the circulation is mediated by a TH transporter other than MCT8, such as LAT1. It would be worth studying if T4 is then transported into the fibrocytes by MCT8, converted into T3 by DIO2 activity, and exported by MCT8 or another TH transporter such as LAT2, and whether T3 enters the target cells in the sensory epithelium by MCT8 and/or LAT1. Another relevant issue to address is whether MCT8 mediates uptake or efflux of TH (40, 41) during cochlear development. The fact that both *Dio3* and *Mct8* expression precede *Dio2* expression could indicate that, at least at early stages, MCT8 cooperates with DIO3 to prevent TH from prematurely binding to TRβ. Lastly, even though TH transporters are known to be involved in bone development (42), the role of TH transport has not been studied in the context of middle ear development.

Our current understanding of the impact of MCT8 deficiency on the hearing capacity of AHDS patients remains limited and incomplete. Hearing loss is not usually a feature of AHDS but has been reported in some patients (4, 43). The relevance of the current findings to human inner ear development should be explored. Another aspect to consider is whether AHDS patients maintain their hearing capacity throughout their entire lifespan or whether they are prone to hearing loss due to deficient TH signalling.

## Vision

TH action is also essential for retinal development and maturation (44). The retina is a light-sensitive tissue layer that contains specialised cells that transform light into electrical signals, ultimately transmitting visual information to the brain. Rod and cone photoreceptors are specialised sensory neurons that initiate phototransduction in the retina. Rods, the most abundant cell type in the retina, mediate dark-adapted vision. Conversely, cones account for just a small fraction of the overall photoreceptor population and mediate colour vision during bright-light conditions. In rodents, cones express different opsin photopigments that are either sensitive to middle (M, green) or short (S, blue) wavelengths (45).

Impaired retinal development and function have been observed in different animal models as a result of developmental hypothyroidism (46), adult-onset hypothyroidism (47), as well as a consequence of deletions in TRs (48), deiodinases (49, 50), and TH transporters (51, 52). Developmental hypothyroidism has been shown to alter the layering of the retina and differentiation of retinal cells (46), while adult hypothyroidism affects opsin expression patterns (47).

Expression pattern studies of TRs in chick revealed that *Thrα1* is expressed in all retinal layers, *Thrβ1* is present at the outer and inner nuclear layers during development and that *Thrβ2* expression is restricted to the outer nuclear layer of the retina containing the developing photoreceptors (53). TRβ2-deficient mice present a decrease in M-cones and an increase in S-opsin cones, indicating that binding of TH to TRβ2 promotes M-cone identity (48). Expression pattern studies of deiodinases, also performed in mice, identified a peak in *Dio3* expression preceding an increase in the expression levels of *Dio2* during retinal development, suggesting opposite patterns of developmental expression for *Dio3* and *Dio2* (50). *Dio2* has been detected in Müller glia (54), in cone photoreceptors in contrast to rods (55), and at the inner nuclear layer (50), with its expression peaking at juvenile stages between P11 and P24 in mice (55). DIO3-deficient mice were shown to lose both M-cones and S-cones, while rod photoreceptors remained intact (49). Interestingly, cone development and differentiation were preserved in mice lacking both DIO3 and DIO2, indicating counterbalancing roles for these deiodinases in regulating TH action (50). All of this could indicate that the binding of T3 to TRβ2 would be essential to promote the correct development of cone photoreceptors and that DIO3 could protect from excessive TH action during this process.

**Figure 2:**
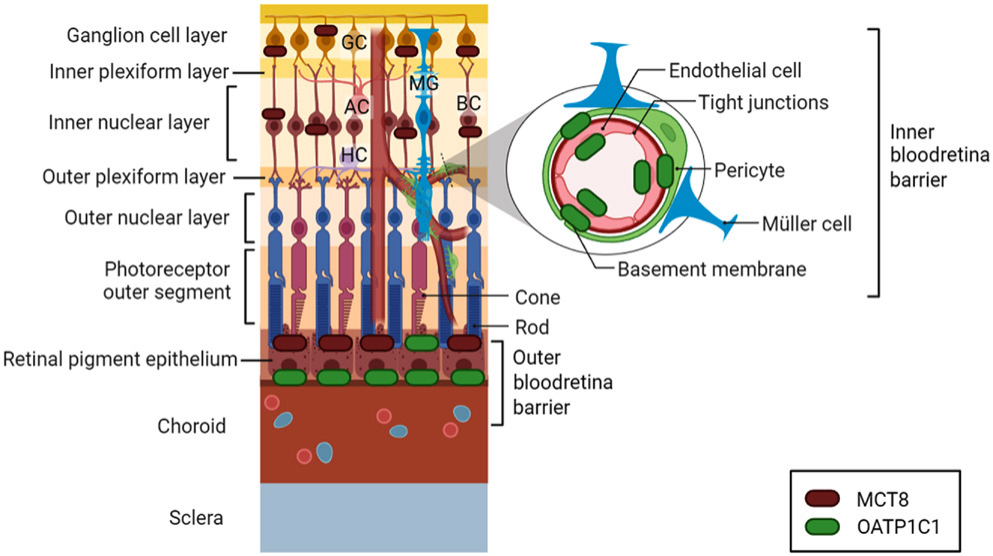
Proposed location of thyroid hormone (TH) transporters in the retina based on current findings. Akanuma *et al.* (56) found OATP1C1 at both at the inner and outer blood–retina barrier, at the retinal pigment epithelium and the inner retinal blood vessels. Henning *et al.* (57) found MCT8 present at the outer blood–retina barrier, at the interface between the retinal pigment epithelium and the photoreceptors. MCT8 was also been found in the inner nuclear and ganglion cell layers, but not expressed at the inner blood–retina barrier. AC (amacrine cell), BC (bipolar cell), GC (ganglion cell), HC (horizontal cell), MG (Müller glia).

There is more limited understanding regarding the transport of TH into the retina. The blood–retina barrier (BRB) consists of two distinct layers: the outer BRB, found at the retinal pigment epithelial (RPE) cell layer, controls the transfer of substances and nutrients from the choroid to the sub-retinal space. In contrast, the inner BRB, similar to the blood–brain barrier, is situated in the inner retinal blood vessels and consists of the endothelial cells lining these vessels. To summarise experimental evidences found in the literature so far, a model for the location of TH transporters in the retina is proposed in Fig. 2. OATP1C1 has been observed at the abluminal and luminal membrane of the inner retinal blood vessels of the inner nuclear layer and inner and outer plexiform layer of the rat retina. OATP1C1 was also observed at the basolateral membrane of the rat RPE (56). MCT8 expression in the early postnatal mouse brain was observed in the interface between the RPE cell layers and the photoreceptors, suggesting that other TH transporters, such as OATP1C1, could regulate the access of TH from the blood to the RPE. Moreover, MCT8 was also identified at cells of the inner nuclear layers and the ganglion cell layer. Contrary to photoreceptors, cells in the inner nuclear layers and the ganglion cell layer are not connected to the RPE, and the access of TH to these cells would be modulated by the inner BRB, where MCT8 expression was not found (57). Henning and Szafranski (57) also found higher MCT8 expression in juvenile mouse retinae in comparison to adults, suggesting a role for MCT8 in the postnatal maturation of the retina. In chicken, MCT8 has been found in the retina from embryonic day 6 (E6) throughout all embryonic development, suggesting a role of MCT8 in the regulation of TH during retinal development (58). Vancamp *et al.* showed that knocking down MCT8 in chick retinal precursor cells during early development leads to reduced proliferation of retinal precursor cells and decreased thickness of the retina; however, a balanced proportion of the different generated cell types suggested no defects in differentiation. Interestingly, while the proportion of rods was unaffected, more S-cones were found at the expense of M-cones (51), resembling findings in TRβ2-deficient mice, although with a milder phenotype. Recent transcriptomic profiling analysis by Rozenblat *et al.* (52) in mct8^−/−^ zebrafish larvae revealed altered expression of vision-related genes, including downregulation of *opn1mw2* (encoding a medium-wavelength sensitive opsin) (52), consistent with findings in MCT8 knockdown chicks. In addition, these authors found a reduction in the number of saccades and pursuits as well as alterations in the activity of pretectal neurons, suggesting that Mct8 deficiency might alter the development and function of these neurons and lead to impairments in conjugate eye movements.

Eye disorders have been documented in some AHDS patients including anisocoria (59, 60) and even blindness (5, 60). Notably, one MCT8-deficient patient developed severe visual impairments during his clinical follow-up (61). However, due to the limited number of documented cases of MCT8 deficiency associated with visual impairments, it is not possible to confirm whether these impairments are direct causes of mutations in MCT8. The data available regarding the role of MCT8 in the retina are very scarce and many questions are still unanswered. For example, even though AHDS patients do not present severe visual impairments, could there be an increased susceptibility to conditions such as colour blindness and impairments in conjugate eye movements? Gathering further insights on the role of MCT8 in the retina will shed more light regarding the potential visual alterations in AHDS.

## Olfaction and taste

TH also plays a role in the development and functioning of olfactory neurons and taste buds (62, 63). Adult-onset hypothyroidism has been shown to disrupt the differentiation of olfactory neurons in the olfactory bulb (OB), leading to decreased thickness of the olfactory epithelium and anosmia (64). TH deficiency also seems to alter taste preference behaviour in adult rats (65).

The olfactory system is formed by olfactory sensory neurons (OSNs) which axons project to the OB, initially through the olfactory nerve layer (ONL) before entering the glomeruli. Within this structure, OSN axons establish synapses with mitral (located in the mitral cell layer, MCL) and tufted cells (located in the external plexiform layer, EPL). Every mitral and tufted cell extends a long axon along the olfactory tract. The olfactory tract distributes these axonal fibres to the cortex of the pyriform lobe, which is the cortical destination for the olfactory pathway (66).

In agreement with the identified role for TH in olfactory neurons, *Thrb* expression has been identified in immature olfactory neurons of chum salmon (67); however, to our knowledge the effects of deletions of TR in olfactory performance have not been assessed. *Dio3* is highly expressed during development in the mouse OB (68) and DIO3-deficient female mice exhibit impaired olfactory function (69). Even though astrocytes are found in all layers of the OB (70), *Dio2* expression has been observed more prominently in the EPL of the rat OB (71). MCT8 has been found to be highly expressed in the anterior olfactory nucleus and in the granular cell layer of the mouse OB (72). Studies exploring the olfactory behaviour in mice lacking MCT8 and OATP1C1 have identified alterations in short-term olfactory memory although this has been associated with reduced adult neurogenesis in the subventricular zone and not with alterations in the OB itself (73).

It is known that THs play an important role in the maturation and specialisation of taste buds (62), but the mechanisms for TH action in these cells have not been explored. Taste buds are composed of type I cells, which are glial-like cells that may transduce salty taste; type II cells that express GPCR receptors and are possibly involved in mediating sweet, umami and bitter tastes; and type III presynaptic cells that are possibly involved transducing sour taste. Type II cells are thought to communicate with type III cells through P2Y adenosine receptors, and ultimately, type III cells release serotonin to afferent neurons to facilitate taste signal transmission to the brain (74). To our knowledge, there are no studies identifying the location or the role of TRs, deiodinases or TH transporters in taste buds. However, the effect of thyroid function in taste has been studied in the context of the burning mouth syndrome, where patients show low levels of TH and high levels of TSH, that lead to hypogeusia and burning sensations (75).

There are no reports of alterations in the smelling capacity or taste in AHDS patients. However, since alterations in these sensory capacities, while significant in their own, may have received less attention compared to the more incapacitating neuromotor and cognitive impairments experienced by these patients, could these might have gone unnoticed?

## Touch

There is very little information regarding the role of TH in mechanoreception and nociception. In one study, Yonkers and Ribera (76) found that, in zebrafish, T4 administration rapidly modulated voltage-gated sodium currents in the mechanoreceptive Rohon–Beard sensory cells, indicating that T4 might be regulating mechanoreception by non-genomic actions.

TH action might also be involved in nociception. It has been described that the sensitivity to nociceptive inhibitory agents, such as adenosine, is increased in response to hypothyroidism, as TH modulate the development, expression and function of adenosine A1 receptors in rats (77). On the other hand, hyperthyroidism in rats has been shown to modify the hydrolysis of AMP to adenosine within synaptosomes of the spinal cord, resulting in either analgesic or hyperalgesic responses depending on the timing of hyperthyroidism onset (78). In addition, TH deficiency during rat intrauterine development seems to reduce the thermal nociceptive threshold and to increase thermal sensitivity, without affecting mechanical nociceptive responses (79).

In view of the findings described previously, and since MCT8 deficiency leads to low T4 circulating levels and brain content, could mechanoreception potentially be affected in AHDS patients? In addition, could defective TH signalling lead to changes in pain perception in these patients?

## Conclusion

Ng *et al.* (80) pointed out that the senses are our window to the world, our connection to our environment, and the foundation of our communication with one another. THs regulate all senses, however, how these might be affected in AHDS patients has not been thoroughly explored. Understanding the relevance of MCT8-mediated TH transport in sensory systems could help to address the phenotypical complexity of the AHDS. In this review, we have gathered data regarding TH action in hearing, vision, smell, taste and touch in different animal models that could suggest how MCT8 deficiency might impact these senses.

One of the main limitations in this area of research is that, while the location and expression of some TH receptors and transporters has been described in the sensory systems, the precision of these findings is limited. For instance, most of the results found in the literature reported the use of antibody-based techniques, which might be affected by the low expression of the proteins and/or the lack of reliable antibodies. Others have used *in situ* hybridisation approaches with low cellular resolution. In consequence, it is not possible to reach definitive and unequivocal conclusions about the exact locations of TH receptors and transporters in the different biological and sensory systems studied. Of interest, very few studies have focused on addressing the mechanisms of TH transport in the sensory systems in the recent years. Future studies using cell-specific and increased sensitive technologies, such us sorting and/or single cell analyses, may help to answer these relevant questions.

In particular, there is not enough data regarding the role of MCT8 in sensory inputs. With this limited data, it is not possible to discern whether the few sensorial defects described in these patients are direct consequence of MCT8 mutations or not. Another issue to consider is that, should these senses be affected, the manifestation of such effects could vary across patients. In addition, the complex TH profile of MCT8-deficient patients characterised by brain hypothyroidism and peripheral hyperthyroidism raises questions about the potential impact of T3 excess on the development of sensory functions, such as touch which relies not only on the central nervous system but also on the peripheral nervous system.

In short, the role of MCT8 in sensory inputs remains an open and interesting question worth exploring. In view of the available data, could MCT8-deficient patients be prone to hearing loss, colour blindness, impairments in conjugate eye movements, dysosmia and dysgeusia, or impaired pain perception? Addressing these potential impairments could ultimately contribute to the well-being of patients.

## Declaration of interest

The authors declare that the study was conducted in the absence of any commercial or financial relationships that could be construed as a potential conflict of interest.

## Funding

This study was supported by grants from the Spanish Ministry of Research and Innovation MCIN/AEI/10.13039/501100011033 and the European Union NextGenerationEU/PRTR (Grant No IJC2020-043543-I) to SB-L, and MCIN/AEI/10.13039/501100011033 and ‘ERDF A way of making Europe’ (grant no. PID2020-113139RB-I00 to AG-F.), a grant from The Sherman Foundation, Australia (grant no. OTR02211 to SB-L and AG-F) and from the Consejo Superior de Investigaciones Científicas, Spain (grant no. 2020AEP044 to AG-F). MG-Y is a recipient of a fellowship from the Programa de Formación de Profesorado (FPU, FPU19/02006), Plan Estatal de I+D+I of the Ministerio de Universidadeshttp://dx.doi.org/10.13039/501100023561, Spain. VV-H is a recipient of a contract from MCIN/AEI/10.13039/501100011033 and by ‘ESF Investing in your future’, Spain (grant no. PRE2018-086185). AG-A is a recipient of a contract FPI-UAM from Universidad Autónoma de Madrid, Spain.

## Author contribution statement

AG-A: writing – original draft; MG-Y: writing – original draft; VV-H: writing – original draft; AM-P: writing – review and editing; AG-F: writing – review and editing; SB-L: writing – review and editing, generation of figures, conceptualisation.

## References

[1] Bernal J Guadaño-Ferraz A & Morte B. Thyroid hormone transporters--functions and clinical implications. Nature Reviews Endocrinology 2015 11 406–417. (10.1038/nrendo.2015.66) 25942657

[2] Brtko J. Thyroid hormone and thyroid hormone nuclear receptors: history and present state of art. Endocrine Regulations 2021 55 103–119. (10.2478/enr-2021-0012) 34020531

[3] Allan W Herndon C & Dudley FC. Some examples of the inheritance of mental deficiency: apparently sex-linked idiocy and microcephaly. American Journal of Mental Deficiency 1944 48 325–334.

[4] Dumitrescu AM Liao XH Best TB Brockmann K & Refetoff S. A novel syndrome combining thyroid and neurological abnormalities is associated with mutations in a monocarboxylate transporter gene. American Journal of Human Genetics 2004 74 168–175. (10.1086/380999) 14661163 PMC1181904

[5] Friesema ECH Grueters A Biebermann H Krude H von Moers A Reeser M Barrett TG Mancilla EE Svensson J Kester MH, *et al.* Association between mutations in a thyroid hormone transporter and severe X-linked psychomotor retardation. Lancet 2004 364 1435–1437. (10.1016/S0140-6736(0417226-7) 15488219

[6] Dumitrescu AM Liao XH Weiss RE Millen K & Refetoff S. Tissue-specific thyroid hormone deprivation and excess in monocarboxylate transporter (mct) 8-deficient mice. Endocrinology 2006 147 4036–4043. (10.1210/en.2006-0390) 16709608

[7] Trajkovic M Visser TJ Mittag J Horn S Lukas J Darras VM Raivich G Bauer K & Heuer H. Abnormal thyroid hormone metabolism in mice lacking the monocarboxylate transporter 8. Journal of Clinical Investigation 2007 117 627–635. (10.1172/JCI28253) 17318265 PMC1797602

[8] López-Espíndola D Morales-Bastos C Grijota-Martínez C Liao XH Lev D Sugo E Verge CF Refetoff S Bernal J & Guadaño-Ferraz A. Mutations of the thyroid hormone transporter MCT8 cause prenatal brain damage and persistent hypomyelination. Journal of Clinical Endocrinology and Metabolism 2014 99 E2799–E2804. (10.1210/jc.2014-2162) 25222753 PMC4255116

[9] Ceballos A Belinchon MM Sanchez-Mendoza E Grijota-Martinez C Dumitrescu AM Refetoff S Morte B & Bernal J. Importance of monocarboxylate transporter 8 for the blood-brain barrier-dependent availability of 3,5,3'-triiodo-L-thyronine. Endocrinology 2009 150 2491–2496. (10.1210/en.2008-1616) 19147674 PMC2671898

[10] Iwayama H Liao XH Braun L Bárez-López S Kaspar B Weiss RE Dumitrescu AM Guadaño-Ferraz A & Refetoff S. Adeno associated virus 9-based gene therapy delivers a functional monocarboxylate Transporter 8, improving thyroid hormone availability to the brain of Mct8-deficient mice. Thyroid 2016 26 1311–1319. (10.1089/thy.2016.0060) 27432638 PMC5036314

[11] Vatine GD Al-Ahmad A Barriga BK Svendsen S Salim A Garcia L Garcia VJ Ho R Yucer N Qian T, *et al.* Modeling psychomotor retardation using iPSCs from MCT8-deficient patients indicates a prominent role for the blood-brain barrier. Cell Stem Cell 2017 20 831–843.e5. (10.1016/j.stem.2017.04.002) 28526555 PMC6659720

[12] van Geest FS Groeneweg S & Visser WE. Monocarboxylate transporter 8 deficiency: update on clinical characteristics and treatment. Endocrine 2021 71 689–695. (10.1007/s12020-020-02603-y) 33650046 PMC8016746

[13] Masnada S Groenweg S Saletti V Chiapparini L Castellotti B Salsano E Visser WE & Tonduti D. Novel mutations in SLC16A2 associated with a less severe phenotype of MCT8 deficiency. Metabolic Brain Disease 2019 34 1565–1575. (10.1007/s11011-019-00464-7) 31332729

[14] Remerand G Boespflug-Tanguy O Tonduti D Touraine R Rodriguez D Curie A Perreton N Des Portes V Sarret C & RMLX/AHDS Study Group. Expanding the phenotypic spectrum of Allan–Herndon–Dudley syndrome in patients with SLC16A2 mutations. Developmental Medicine and Child Neurology 2019 61 1439–1447. (10.1111/dmcn.14332) 31410843

[15] DeLong GR Stanbury JB & Fierro-Benitez R. Neurological signs in congenital iodine-deficiency disorder (endemic cretinism). Developmental Medicine and Child Neurology 1985 27 317–324. (10.1111/j.1469-8749.1985.tb04542.x) 4018426

[16] Rovet J Walker W Bliss B Buchanan L & Ehrlich R. Long-term sequelae of hearing impairment in congenital hypothyroidism. Journal of Pediatrics 1996 128 776–783. (10.1016/s0022-3476(9670329-3) 8648536

[17] Fettiplace R. Hair cell transduction, tuning, and synaptic transmission in the mammalian cochlea. Comprehensive Physiology 2017 7 1197–1227. (10.1002/cphy.c160049) 28915323 PMC5658794

[18] Deol MS. The role of thyroxine in the differentiation of the organ of Corti. Acta Otolaryngologica 1976 81 429–435. (10.3109/00016487609107497) 944989

[19] Hébert R Langlois JM & Dussault JH. Permanent defects in rat peripheral auditory function following perinatal hypothyroidism: determination of a critical period. Brain Research 1985 355 161–170. (10.1016/0165-3806(8590037-9) 4084772

[20] Knipper M Richardson G Mack A Müller M Goodyear R Limberger A Rohbock K Köpschall I Zenner HP & Zimmermann U. Thyroid hormone-deficient period prior to the onset of hearing is associated with reduced levels of beta-tectorin protein in the tectorial membrane: implication for hearing loss. Journal of Biological Chemistry 2001 276 39046–39052. (10.1074/jbc.M103385200) 11489885

[21] Rusch A Ng L Goodyear R Oliver D Lisoukov I Vennstrom B Richardson G Kelley MW & Forrest D. Retardation of cochlear maturation and impaired hair cell function caused by deletion of all known thyroid hormone receptors. Journal of Neuroscience 2001 21 9792–9800. (10.1523/JNEUROSCI.21-24-09792.2001) 11739587 PMC6763054

[22] Jones I Srinivas M Ng L & Forrest D. The thyroid hormone receptor beta gene: structure and functions in the brain and sensory systems. Thyroid 2003 13 1057–1068. (10.1089/105072503770867228) 14651789

[23] Ng L Cordas E Wu X Vella KR Hollenberg AN & Forrest D. Age-related hearing loss and degeneration of cochlear hair cells in mice lacking thyroid hormone receptor β1. Endocrinology 2015 156 3853–3865. (10.1210/en.2015-1468) 26241124 PMC4588828

[24] Ng L Goodyear RJ Woods CA Schneider MJ Diamond E Richardson GP Kelley MW Germain DL Galton VA & Forrest D. Hearing loss and retarded cochlear development in mice lacking type 2 iodothyronine deiodinase. PNAS 2004 101 3474–3479. (10.1073/pnas.0307402101) 14993610 PMC373486

[25] Ng L Hernandez A He W Ren T Srinivas M Ma M Galton VA St Germain DL & Forrest D. A protective role for type 3 deiodinase, a thyroid hormone-inactivating enzyme, in cochlear development and auditory function. Endocrinology 2009 150 1952–1960. (10.1210/en.2008-1419) 19095741 PMC2659284

[26] Sharlin DS Ng L Verrey F Visser TJ Liu Y Olszewski RT Hoa M Heuer H & Forrest D. Deafness and loss of cochlear hair cells in the absence of thyroid hormone transporters Slc16a2 (Mct8) and Slc16a10 (Mct10). Scientific Reports 2018 8 4403. (10.1038/s41598-018-22553-w) 29535325 PMC5849681

[27] Bradley DJ Towle HC & Young WS 3rd. Alpha and beta thyroid hormone receptor (TR) gene expression during auditory neurogenesis: evidence for TR isoform-specific transcriptional regulation in vivo. PNAS 1994 91 439–443. (10.1073/pnas.91.2.439) 8290545 PMC42964

[28] Lautermann J & ten Cate WJ. Postnatal expression of the alpha-thyroid hormone receptor in the rat cochlea. Hearing Research 1997 107 23–28. (10.1016/s0378-5955(9700014-2) 9165343

[29] Forrest D Erway LC Ng L Altschuler R & Curran T. Thyroid hormone receptor beta is essential for development of auditory function. Nature Genetics 1996 13 354–357. (10.1038/ng0796-354) 8673137

[30] Winter H Rüttiger L Müller M Kuhn S Brandt N Zimmermann U Hirt B Bress A Sausbier M Conscience A, *et al.* Deafness in TRbeta mutants is caused by malformation of the tectorial membrane. Journal of Neuroscience 2009 29 2581–2587. (10.1523/JNEUROSCI.3557-08.2009) 19244534 PMC2748340

[31] Rüsch A Erway LC Oliver D Vennström B & Forrest D. Thyroid hormone receptor beta-dependent expression of a potassium conductance in inner hair cells at the onset of hearing. PNAS 1998 95 15758–15762. (10.1073/pnas.95.26.15758) 9861043 PMC28117

[32] Ng L Rüsch A Amma LL Nordström K Erway LC Vennström B & Forrest D. Suppression of the deafness and thyroid dysfunction in Thrb-null mice by an independent mutation in the Thra thyroid hormone receptor α gene. Human Molecular Genetics 2001 10 2701–2708. (10.1093/hmg/10.23.2701) 11726557

[33] Affortit C Blanc F Nasr J Ceccato JC Markossian S Guyot R Puel JL Flamant F & Wang J. A disease-associated mutation in thyroid hormone receptor α1 causes hearing loss and sensory hair cell patterning defects in mice. Science Signaling 2022 15 eabj4583. (10.1126/scisignal.abj4583) 35700264

[34] Cordas EA Ng L Hernandez A Kaneshige M Cheng SY & Forrest D. Thyroid hormone receptors control developmental maturation of the middle ear and the size of the ossicular bones. Endocrinology 2012 153 1548–1560. (10.1210/en.2011-1834) 22253431 PMC3281545

[35] Bárez-López S Montero-Pedrazuela A Bosch-García D Venero C & Guadaño-Ferraz A. Increased anxiety and fear memory in adult mice lacking type 2 deiodinase. Psychoneuroendocrinology 2017 84 51–60. (10.1016/j.psyneuen.2017.06.013) 28654773

[36] Sharlin DS Visser TJ & Forrest D. Developmental and cell-specific expression of thyroid hormone transporters in the mouse cochlea. Endocrinology 2011 152 5053–5064. (10.1210/en.2011-1372) 21878515 PMC3230046

[37] Peeleman N Verdoodt D Ponsaerts P & Van Rompaey V. On the role of fibrocytes and the extracellular matrix in the physiology and pathophysiology of the spiral ligament. Frontiers in Neurology 2020 11 580639. (10.3389/fneur.2020.580639) 33193034 PMC7653186

[38] Campos-Barros A Amma LL Faris JS Shailam R Kelley MW & Forrest D. Type 2 iodothyronine deiodinase expression in the cochlea before the onset of hearing. PNAS 2000 97 1287–1292. (10.1073/pnas.97.3.1287) 10655523 PMC15599

[39] Espino Guarch M Font-Llitjós M Murillo-Cuesta S Errasti-Murugarren E Celaya AM Girotto G Vuckovic D Mezzavilla M Vilches C Bodoy S, *et al.* Mutations in L-type amino acid transporter-2 support SLC7A8 as a novel gene involved in age-related hearing loss. eLife 2018 7. (10.7554/eLife.31511) PMC5811215 29355479

[40] Friesema EC Ganguly S Abdalla A Manning Fox JE Halestrap AP & Visser TJ. Identification of monocarboxylate transporter 8 as a specific thyroid hormone transporter. Journal of Biological Chemistry 2003 278 40128–40135. (10.1074/jbc.M300909200) 12871948

[41] Friesema ECH Jansen J Jachtenberg JW Visser WE Kester MHA & Visser TJ. Effective cellular uptake and efflux of thyroid hormone by human monocarboxylate Transporter 10. Molecular Endocrinology 2008 22 1357–1369. (10.1210/me.2007-0112) 18337592 PMC5419535

[42] Bassett JH & Williams GR. Role of thyroid hormones in skeletal development and bone maintenance. Endocrine Reviews 2016 37 135–187. (10.1210/er.2015-1106) 26862888 PMC4823381

[43] Gagliardi L Nataren N Feng J Schreiber AW Hahn CN Conwell LS Coman D & Scott HS. Allan-Herndon-Dudley syndrome with unusual profound sensorineural hearing loss. American Journal of Medical Genetics 2015 167A 1872–1876. (10.1002/ajmg.a.37075) 25850411

[44] Forrest D Reh TA & Rüsch A. Neurodevelopmental control by thyroid hormone receptors. Current Opinion in Neurobiology 2002 12 49–56. (10.1016/s0959-4388(0200289-1) 11861164

[45] Yang F Ma H & Ding X-Q. Chapter fourteen - thyroid hormone signaling in retinal development, survival, and disease. In Vitamins and Hormones, pp. 333–349. Litwack G, Ed. New York: Academic Press 2018. 10.1016/bs.vh.2017.05.001 29407441

[46] Sevilla-Romero E Muñoz A & Pinazo-Durán MD. Low thyroid hormone levels impair the perinatal development of the rat retina. Ophthalmic Research 2002 34 181–191. (10.1159/000063885) 12297689

[47] Glaschke A Weiland J Del Turco D Steiner M Peichl L & Glösmann M. Thyroid hormone controls cone opsin expression in the retina of adult rodents. Journal of Neuroscience 2011 31 4844–4851. (10.1523/JNEUROSCI.6181-10.2011) 21451022 PMC6622973

[48] Ng L Hurley JB Dierks B Srinivas M Saltó C Vennström B Reh TA & Forrest D. A thyroid hormone receptor that is required for the development of green cone photoreceptors. Nature Genetics 2001 27 94–98. (10.1038/83829) 11138006

[49] Ng L Lyubarsky A Nikonov SS Ma M Srinivas M Kefas B St Germain DL Hernandez A Pugh EN & Forrest D. Type 3 deiodinase, a thyroid-hormone-inactivating enzyme, controls survival and maturation of cone photoreceptors. Journal of Neuroscience 2010 30 3347–3357. (10.1523/JNEUROSCI.5267-09.2010) 20203194 PMC2843520

[50] Ng L Liu H St. Germain DL Hernandez A & Forrest D. Deletion of the thyroid hormone–activating Type 2 deiodinase rescues cone photoreceptor degeneration but not deafness in mice lacking Type 3 deiodinase. Endocrinology 2017 158 1999–2010. (10.1210/en.2017-00055) 28324012 PMC5460942

[51] Vancamp P Bourgeois NMA Houbrechts AM & Darras VM. Knockdown of the thyroid hormone transporter MCT8 in chicken retinal precursor cells hampers early retinal development and results in a shift towards more UV/blue cones at the expense of green/red cones. Experimental Eye Research 2019 178 135–147. (10.1016/j.exer.2018.09.018) 30273578

[52] Rozenblat R Tovin A Zada D Lebenthal-Loinger I Lerer-Goldshtein T & Appelbaum L. Genetic and neurological deficiencies in the visual system of mct8 mutant zebrafish. International Journal of Molecular Sciences 2022 23. (10.3390/ijms23052464) PMC8910067 35269606

[53] Sjöberg M Vennström B & Forrest D. Thyroid hormone receptors in chick retinal development: differential expression of mRNAs for alpha and N-terminal variant beta receptors. Development 1992 114 39–47. (10.1242/dev.114.1.39) 1576965

[54] Wei M Sun Y Li S Chen Y Li L Fang M Shi R Tong D Chen J Ma Y, *et al.* Single-cell profiling reveals Müller glia coordinate retinal intercellular communication during light/dark adaptation via thyroid hormone signaling. Protein and Cell 2023 14 603–617. (10.1093/procel/pwad007) 36930538 PMC10392031

[55] Sawant OB Horton AM Zucaro OF Chan R Bonilha VL Samuels IS & Rao S. The circadian clock gene Bmal1 controls thyroid hormone-mediated spectral identity and cone photoreceptor function. Cell Reports 2017 21 692–706. (10.1016/j.celrep.2017.09.069) 29045837 PMC5647869

[56] Akanuma S Hirose S Tachikawa M & Hosoya K. Localization of organic anion transporting polypeptide (Oatp) 1a4 and Oatp1c1 at the rat blood-retinal barrier. Fluids and Barriers of the CNS 2013 10 29. (10.1186/2045-8118-10-29) 24083450 PMC3850135

[57] Henning Y & Szafranski K. Age-dependent changes of monocarboxylate Transporter 8 availability in the postnatal murine retina. Frontiers in Cellular Neuroscience 2016 10 205. (10.3389/fncel.2016.00205) 27616981 PMC4999454

[58] Geysens S Ferran JL Van Herck SL Tylzanowski P Puelles L & Darras VM. Dynamic mRNA distribution pattern of thyroid hormone transporters and deiodinases during early embryonic chicken brain development. Neuroscience 2012 221 69–85. (10.1016/j.neuroscience.2012.06.057) 22771619

[59] Bialer MG Lawrence L Stevenson RE Silverberg G Williams MK Arena JF Lubs HA & Schwartz CE. Allan-Herndon-Dudley syndrome: clinical and linkage studies on a second family. American Journal of Medical Genetics 1992 43 491–497. (10.1002/ajmg.1320430173) 1605231

[60] Schwartz CE & Stevenson RE. The MCT8 thyroid hormone transporter and Allan-Herndon-Dudley syndrome. Best Practice and Research. Clinical Endocrinology and Metabolism 2007 21 307–321. (10.1016/j.beem.2007.03.009) 17574010 PMC2094733

[61] Filho HC Marui S Manna TD Brust ES Radonsky V Kuperman H Dichtchekenian V Setian N & Damiani D. Novel mutation in MCT8 gene in a Brazilian boy with thyroid hormone resistance and severe neurologic abnormalities. Arquivos Brasileiros de Endocrinologia e Metabologia 2011 55 60–66. (10.1590/s0004-27302011000100008) 21468521

[62] McConnell RJ Menendez CE Smith FR Henkin RI & Rivlin RS. Defects of taste and smell in patients with hypothyroidism. American Journal of Medicine 1975 59 354–364. (10.1016/0002-9343(7590394-0) 1163545

[63] Deniz F Ay SA Salihoglu M Kurt O Baskoy K Altundag A Tekeli H Yonem A & Hummel T. Thyroid hormone replacement therapy improves olfaction and taste sensitivity in primary hypothyroid patients: A prospective randomised clinical trial. Experimental and Clinical Endocrinology and Diabetes 2016 124 562–567. (10.1055/s-0042-108446) 27437913

[64] Mackay-Sim A & Beard MD. Hypothyroidism disrupts neural development in the olfactory epithelium of adult mice. Brain Research 1987 433 190–198. (10.1016/0165-3806(8790023-x) 3690332

[65] Brosvic GM Doty RL Rowe MM Harron A & Kolodiy N. Influences of hypothyroidism on the taste detection performance of rats: a signal detection analysis. Behavioral Neuroscience 1992 106 992–998. (10.1037//0735-7044.106.6.992) 1472298

[66] Imamura F Ito A & LaFever BJ. Subpopulations of projection neurons in the olfactory bulb. Frontiers in Neural Circuits 2020 14 561822. (10.3389/fncir.2020.561822) 32982699 PMC7485133

[67] Kudo H Eto A Abe T & Mochida K. Detection and localization of the thyroid hormone receptor beta mRNA in the immature olfactory receptor neurons of chum salmon. Heliyon 2018 4 e00744. (10.1016/j.heliyon.2018.e00744) 30148220 PMC6106697

[68] Martinez ME Charalambous M Saferali A Fiering S Naumova AK St Germain D Ferguson-Smith AC & Hernandez A. Genomic imprinting variations in the mouse type 3 deiodinase gene between tissues and brain regions. Molecular Endocrinology 2014 28 1875–1886. (10.1210/me.2014-1210) 25232934 PMC4213365

[69] Stohn JP Martinez ME Zafer M López-Espíndola D Keyes LM & Hernandez A. Increased aggression and lack of maternal behavior in Dio3-deficient mice are associated with abnormalities in oxytocin and vasopressin systems. Genes, Brain, and Behavior 2018 17 23–35. (10.1111/gbb.12400) 28715127 PMC5771999

[70] Bailey MS & Shipley MT. Astrocyte subtypes in the rat olfactory bulb: morphological heterogeneity and differential laminar distribution. Journal of Comparative Neurology 1993 328 501–526. (10.1002/cne.903280405) 8429132

[71] Guadaño-Ferraz A Obregón MJ St Germain DL & Bernal J. The type 2 iodothyronine deiodinase is expressed primarily in glial cells in the neonatal rat brain. PNAS 1997 94 10391–10396. (10.1073/pnas.94.19.10391) 9294221 PMC23373

[72] Heuer H Maier MK Iden S Mittag J Friesema EC Visser TJ & Bauer K. The monocarboxylate transporter 8 linked to human psychomotor retardation is highly expressed in thyroid hormone-sensitive neuron populations. Endocrinology 2005 146 1701–1706. (10.1210/en.2004-1179) 15661862

[73] Luongo C Butruille L Sébillot A Le Blay K Schwaninger M Heuer H Demeneix BA & Remaud S. Absence of both thyroid hormone transporters MCT8 and OATP1C1 impairs neural stem cell fate in the adult mouse subventricular zone. Stem Cell Reports 2021 16 337–353. (10.1016/j.stemcr.2020.12.009) 33450189 PMC7878696

[74] Gravina SA Yep GL & Khan M. Human biology of taste. Annals of Saudi Medicine 2013 33 217–222. (10.5144/0256-4947.2013.217) 23793421 PMC6078535

[75] Femiano F Gombos F Esposito V Nunziata M & Scully C. Burning mouth syndrome (BMS): evaluation of thyroid and taste. Medicina Oral, Patologia Oral y Cirugia Bucal 2006 11 E22–E25. 16388288

[76] Yonkers MA & Ribera AB. Sensory neuron sodium current requires nongenomic actions of thyroid hormone during development. Journal of Neurophysiology 2008 100 2719–2725. (10.1152/jn.90801.2008) 18799597 PMC2585397

[77] Braganhol E Bruno AN Bavaresco L Barreto-Chaves ML Sarkis JJ & Battastini AM. Neonatal hypothyroidism affects the adenine nucleotides metabolism in astrocyte cultures from rat brain. Neurochemical Research 2006 31 449–454. (10.1007/s11064-006-9041-y) 16758352

[78] Bruno AN Fontella FU Crema LM Bonan CD Dalmaz C Barreto-Chaves ML & Sarkis JJ. Hyperthyroidism changes nociceptive response and ecto-nucleotidase activities in synaptosomes from spinal cord of rats in different phases of development. Comparative Biochemistry and Physiology 2005 140 111–116. (10.1016/j.cbpb.2004.11.007) 15664319

[79] Alves IG da Cruz KM Mota CM de Santana DS Gaujac DP de Carvalho VC Reis LC Sluka KA Quintans-Junior LJ Antoniolli AR, *et al.* Experimental hypothyroidism during pregnancy affects nociception and locomotor performance of offspring in rats. European Journal of Pain 2013 17 1291–1298. (10.1002/j.1532-2149.2013.00306.x) 23536325

[80] Ng L Kelley MW & Forrest D. Making sense with thyroid hormone--the role of T(3) in auditory development. Nature Reviews Endocrinology 2013 9 296–307. (10.1038/nrendo.2013.58) 23529044

